# Analysis of risk factors for postoperative complications in non-small cell lung cancer: comparison with the Japanese National Clinical Database risk calculator

**DOI:** 10.1186/s12893-022-01628-6

**Published:** 2022-05-14

**Authors:** Nozomu Motono, Masahito Ishikawa, Shun Iwai, Aika Yamagata, Yoshihito Iijima, Hidetaka Uramoto

**Affiliations:** Department of Thoracic Surgery, Kanazawa Medical University, 1-1 Daigaku, Uchinada, Ishikawa 920-0293 Japan

**Keywords:** Postoperative complication, Risk factor, Non-small cell lung cancer

## Abstract

**Background:**

Although the risk calculator of the National Clinical Database (RC-NCD) has been widely used to predict the occurrence of mortality and major morbidity in Japan, it has not been demonstrated whether a correlation between the calculated RC-NCD risk score and the actual occurrence of mortality and severe morbidity exists.

**Methods:**

The clinical data of 585 patients who underwent pulmonary resection for non-small cell lung cancer were collected, and the risk factors for postoperative morbidity were analyzed to verify the validity of the RC-NCD.

**Results:**

The coexistence of asthma (p = 0.02), nutrition lymphocyte ratio (p = 0.04), and pulmonary lobe (p < 0.01) were significant risk factors for postoperative morbidity in the present study, and the percent-predicted vital capacity (p < 0.01), pulmonary lobe (p = 0.03), and type of operative procedure (p = 0.01) were significant risk factors for severe postoperative morbidity. Furthermore, in patients received lobectomy, coexistence of asthma (p = 0.01) and pulmonary lobe (p < 0.01) were identified as significant risk factors for postoperative morbidity. Meanwhile, male sex (p = 0.01), high BMI (p < 0.01), low vital capacity (p = 0.04), and pulmonary lobe (p = 0.03) were identified as significant risk factors for severe postoperative morbidity.

**Conclusions:**

Given that the pulmonary lobe was a significant risk factor for postoperative morbidity in patients received pulmonary resection and for severe postoperative morbidity in patients received lobectomy, the RC-NCD for postoperative morbidity needs to be modified according to high-risk lobes.

*Trial registration:* The Institutional Review Board of Kanazawa Medical University approved the protocol of this retrospective study (approval number: I392), and written informed consent was obtained from all patients.

## Introduction

Lung cancer is the leading cause of cancer-related mortality worldwide [1]. The incidence of postoperative complications associated with pulmonary resection for non-small-cell lung cancer (NSCLC) has been reported to be 9–37% [2–4]. Furthermore, the incidence of postoperative complications associated with lobectomy is 10–50% and has been observed to be higher in the elderly than in younger individuals [5]. Several postoperative complications can occur after pulmonary resection, commonly including air leakage, pneumonia, atelectasis, and arrhythmia. The incidence of postoperative pulmonary complications after pulmonary resection has been reported to be 6–30% [6–10]. Age, smoking history, and chronic obstructive pulmonary disease (COPD) are considered significant risk factors for postoperative pulmonary complications.

**Fig. 1:**
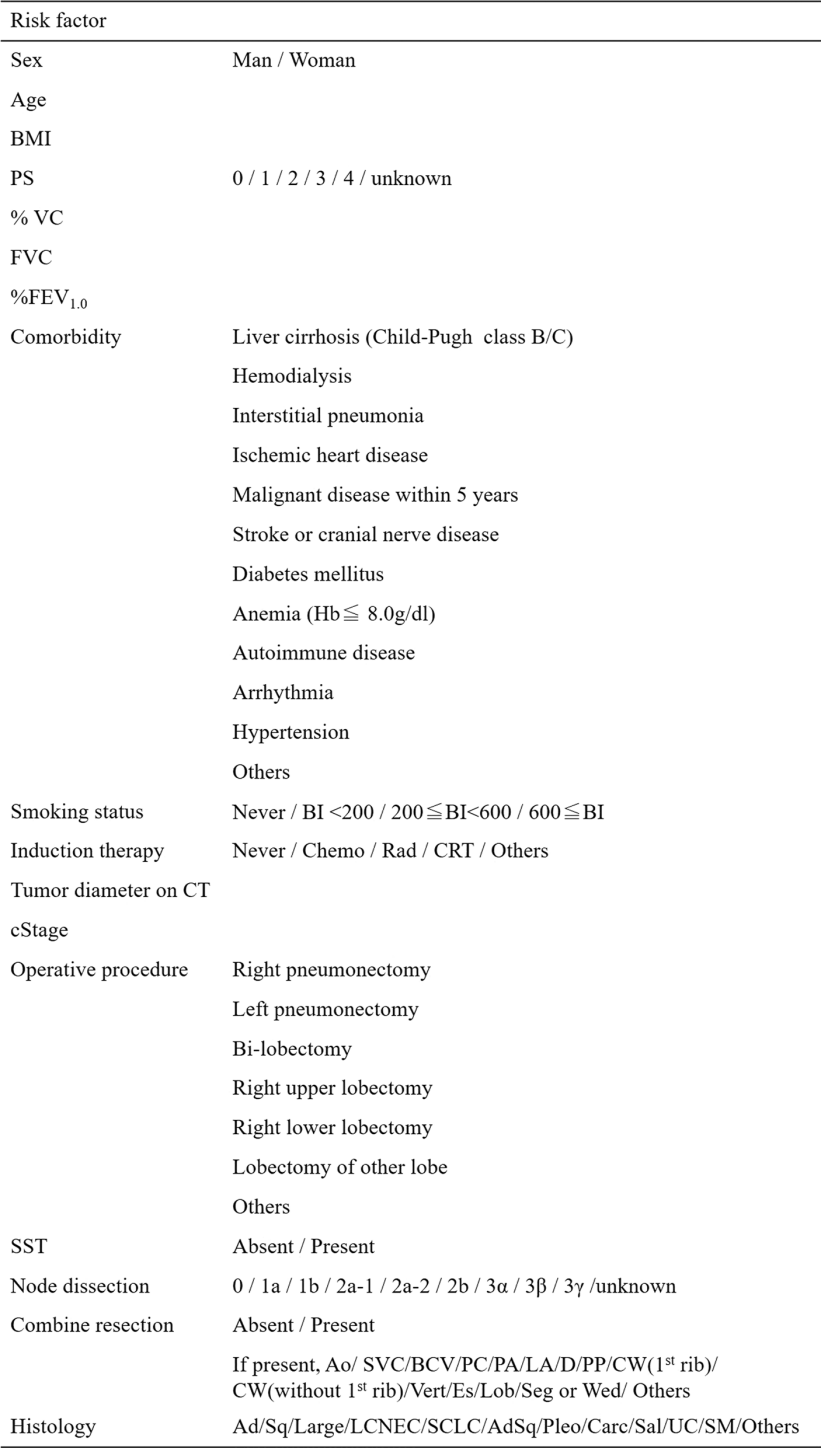
Risk factors of the risk calculator of the national clinical database in Japan. *BMI* body mass index, *PS* performance status, *VC* vital capacity, *%FEV*_*1.0*_ forced expiratory volume % in one second, *Hb* hemoglobin, *BI* Brinkman index, *Chemo* chemotherapy, *Rad* radiation, *CRT* chemoradiotherapy, *CT* computed tomography, *SST*: superior sulcus tumor, *Ao* aorta, *SVC* supra vena cava, *BCV* brachiocephalic vein, *PC* pericardium, *PA* pulmonary artery, *LA* left atrium, *D* diaphragm, *PP* parietal pleura, *CW* chest wall, *Vert* vertebra, *Es* esophagus, *Lob* other pulmonary lobe, *Seg* segmentectomy, *Wed* wedge resection, *Ad* adenocarcinoma, *Sq* squamous cell carcinoma, *Large* large cell carcinoma, *LCNEC* large cell neuroendocrine carcinoma, *SCLC* small cell lung cancer, *AdSq* adenosquamous cell carcinoma, *Pleo* pleomorphic carcinoma, *Carc* carcinoid, *Sal* salivary gland cancer, *UC* unclassifiable cancer, *SM* simultaneous multiple cancer

In 2011, the National Clinical Database (NCD) of Japan began an annual web-based data collection registration system. Data on 1.6 million surgical procedures from more than 4,000 hospitals were collected [11]. The risk calculator system was established based on a model of lung cancer surgery risk derived from the NCD of 78,594 patients from the years 2014 to 2015 [12]. In this study, mortality and the combination of mortality and major morbidity were analyzed. Operative mortality was defined as death within 30 days after surgery, and major morbidity was defined according to the Society of Thoracic Surgeons General Thoracic Database (STS GTDB) [13, 14]. Although the risk calculator of NCD (RC-NCD) has been widely used to predict the occurrence of mortality and major morbidity in Japan (Fig. 1), whether or not there is a correlation between the risk score calculated by the RC-NCD and the actual occurrence of mortality and severe morbidity has not been demonstrated.

In the present study, we retrospectively evaluated the risk factors for mortality and morbidity after pulmonary resection in patients with NCSLC and verified the validity of the RC-NCD.

## Materials and methods

### Patients

**Fig. 2:**
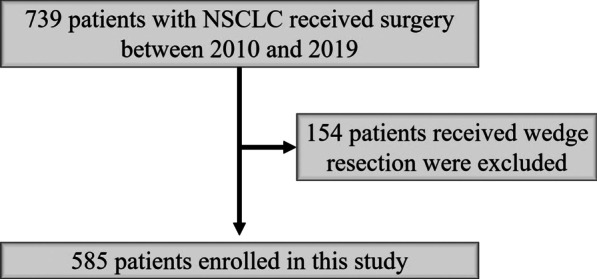
Patient flow diagram

Seven hundred and thirty-nine NSCLC patients were received pulmonary resection at Kanazawa Medical University between January 2010 and March 2019, and 154 patients who underwent wedge resection were excluded. Therefore, 585 patients were enrolled in this retrospective study (Fig. 2).

Data including clinical factors, such as the sex, age, comorbidities, smoking history, body mass index (BMI), carcinoembryonic antigen (CEA) levels, respiratory function, prognostic nutritional index (PNI), neutrophil-to-lymphocyte ratio (NLR), tumor diameter on computed tomography (CT), lobe involvement in lung cancer, clinical and pathological stage, and histology, were collected. The following comorbidities were included, according to the factors of the RC-NCD: malignant disease, hypertension, diabetes mellitus, angina pectoris, COPD, cerebral infarction, arrhythmia, interstitial lung disease, asthma, autoimmune disease, and chronic renal failure. Respiratory function parameters, such as the percent-predicted vital capacity (%VC) and forced expiratory volume in 1 s as a percentage of forced vital capacity (FEV_1_%), were collected. The PNI, calculated from the serum albumin level and total lymphocyte count, is a simple and useful indicator of one’s immune-nutritional status [15]. The NLR was defined as the ratio of the neutrophil to the lymphocyte count, functioning as a parameter of systemic inflammation and stress in critically ill surgical and medical patients [16]. The smoking history was assessed using the Brinkman index, which is calculated by multiplying the number of cigarettes smoked per day by the number of years the subject has been smoking [17].

### Operative factors

The operative approach was divided into three categories: video-assisted thoracic surgery (VATS), robot-assisted thoracic surgery (RATS), and thoracotomy. The operative procedure was divided into eight categories: wedge resection, segmentectomy, lobectomy, sleeve lobectomy, lobectomy combined with segmentectomy, lobectomy combined with chest wall resection, bi-lobectomy, and pneumonectomy.

### Postoperative complications

Postoperative complications were categorized into five grades according to the Clavien–Dindo classification system. Established in 1992, it is a simple and feasible grading system for all types of postoperative complications [18]. In 2004, it was modified to allow for the grading of life-threatening complications and long-term disability caused by a complication [19]. This revised version defined five grades of severity with subgrades (grades I, II, IIIa, IIIb, IVa, IVb, and V), and the suffix “d” (for “disability”) is used to denote any postoperative impairment. This modified version of the Clavien–Dindo classification has been widely used in clinical practice. Severe morbidity was defined according to the classification of RC-NCD, which was based on the STS GTSD risk model and included the following: respiratory failure, interstitial pneumonia, tracheobronchial fistula, pulmonary thromboembolism, pneumonia, redo surgery, myocardial infarction, arrhythmia requiring therapy, renal failure, postoperative bleeding, and chylothorax [12, 13]. Furthermore, we added air leakage requiring therapy, atelectasis, asthma attacks, and cerebral infarction as postoperative morbidities.

### Statistical analyses

Pearson’s chi-squared test of independence was used to compare the frequencies of the variables. Risk factors related to postoperative complications were analyzed using a logistic regression analysis. Factors showing significant differences in the univariate analysis were included in the multivariate analysis. All statistical analyses were two-sided, and statistical significance was set at p < 0.05. Statistical analyses were conducted using the JMP software program (version 13.2; SAS Institute Inc., Cary, NC, USA).

The present study was conducted in accordance with the principles of the Declaration of Helsinki. The Institutional Review Board of Kanazawa Medical University approved the protocol (approval number: I392), and written informed consent was obtained from all patients.

## Results

### Patient characteristics, operative factors, postoperative morbidity, and mortality

The clinical characteristics of the 585 patients are shown in Table 1. Altogether, 333 patients had comorbidities, including 81 patients with the history of other malignant disease in addition to NSCLC (16 with colon cancer, 6 with rectal cancer, 14 with gastric cancer, 2 with esophageal cancer, 12 with breast cancer, 8 with prostate cancer, 9 with bladder cancer, 3 with gallbladder cancer, 7 with thyroid cancer, 3 with renal cancer, 4 with laryngeal cancer, 3 with pharyngeal cancer, and 5 with lymphoma; duplication occurred in some patients), 92 patients with hypertension, 81 with diabetes mellitus, 37 with angina pectoris, 35 with COPD, 24 with cerebral infarction, 20 with arrhythmia (18 with atrial fibrillation, 1 with paroxysmal supraventricular tachycardia, and 1 with atrioventricular block), 9 with interstitial lung disease, 20 with asthma, 15 with autoimmune disease, and 7 with chronic renal failure.

**Table 1:** Patient characteristics and perioperative data

Variables	
Sex (man / woman)	364 / 221
Age, medial, range (y)	69 (34–92)
Comorbidity	333 (56.9%)
Malignant disease	81 (13.8%)
Hypertension	92 (15.7%)
Diabetes mellitus	81 (13.8%)
Angina pectoris	37 (6.3%)
COPD	35 (5.9%)
Cerebral infarction	24 (4.1%)
Arrhythmia	20 (3.4%)
Interstitial lung disease	9 (1.5%)
Asthma	20 (3.4%)
Autoimmune disease	15 (2.5%)
Chronic renal failure	7 (1.1%)
Body mass index, median (range)	22.8 (14.3–36.6)
CEA, median, range (ng/ml)	3.4 (0.5–306)
Brinkman index, median (range)	600 (0–3600)
PNI, median (range)	50.0 (26.9–67.5)
NLR, median (range)	2.14 (0.66–12.65)
%VC, median (range)	111 (45.7–184.2)
FEV1%, median (range)	73.4 (30.5–99.5)
Lobe of lung cancer (RU / RM / RL / LU / LL)	179 / 37 / 137 / 139 / 93
pStage (0 / IA / IB / IIA / IIB / IIIA / IIIB / IV / yIA / yIIA)	32 / 284 / 107 / 43 / 57 / 51 / 3 / 2 / 5 / 1
Histological type (Ad / Sq / LCNEC / AdSq / Pleo / Large / Carci)	441 / 108 / 17 / 9 / 4 / 3 / 3
Operative approach (Open / VATS / RATS)	71 / 662 / 6
Location of Lobectomy (RU / RM / RL / LU / LL)	175 / 36 / 106 / 108 / 76
Operative procedure (Seg / Lob / Lob + CW / Lob + Seg / Sleeve Lob / Bilob / Pneumo)	68 / 473 / 9 / 3 / 4 / 10 / 18
Morbidity	176 (30.0%)
Air leakage	81 (13.8%)
Arrhythmia	44 (7.5%)
Atelectasis	19 (3.2%)
Pneumonia	12 (2.0%)
Attack of asthma	4 (0.6%)
Cerebral infarction	3 (0.5%)
Chylothorax	2 (0.3%)
Broncho-pleural fistula	2 (0.3%)
Clavien–Dindo grade (0 / I / II / IIIa / IIIb)	409 / 1 / 71 / 99 / 5
Mortality	1 (0.1%)
Mortality or severe morbidity	67 (11.4%)
Tracheal or bronchial fistula	2 (0.3%)
Respiratory disfunction	4 (0.6%)
NCD risk score	
Mortality, median (range) (%)	0.2 (0–10.6)
Mortality or severe morbidity, median (range) (%)	4.6 (1–39.3)
Tracheal or bronchial fistula, median (range) (%)	0.2 (0–8.1)
Respiratory disfunction, median (range) (%)	0.2 (0–8.2)

*COPD* chronic obstructive pulmonary disease, *CEA* carcinoembryonic antigen, *PNI* prognostic nutritional index, *NLR* neutrophil-to-lymphocyte ratio, *VC* vital capacity, *FEV*_*1*_*%* forced expiratory volume % in one second, *RU* right upper, *RM* right middle, *RL* right lower, *LU* left upper, *LL* left lower, *p* pathological, *y* yield to treatment, *Ad* adenocarcinoma, *Sq* squamous cell carcinoma, *LCNEC* large cell neuroendocrine carcinoma, *AdSq* adenosquamous cell carcinoma, *Large* large cell carcinoma, *Carci* carcinoid, *Open* open thoracotomy, *VATS* video-assisted thoracic surgery, *RATS* robotic-assisted thoracic surgery, *RU* right upper, *RM* right middle, *RL* right lower, *LU* left upper, *LL* left lower, *RU* right upper, *RM* right middle, *RL* right lower, *LU* left upper, *LL* left lower, *Seg* segmentectomy, *Lob* lobectomy, *CW* chest wall resection, *Bilob* bi-lobectomy, *Pneumo* pneumonectomy, *NCD* national clinical database

Postoperative morbidities were observed in 176 patients (30.0%). Clavien–Dindo grade I complication was noted in 1 patient, grade II in 71, grade IIIa in 99, and grade IIIb in 5. Air leakage occurred in 81 patients, arrhythmia in 44 (atrial fibrillation in 37, paroxysmal supraventricular tachycardia in 3, ventricular tachycardia in 3, and sick sinus syndrome in 1), atelectasis in 19, pneumonia in 12, asthma attacks in 4, cerebral infarction in 3, chylothorax in 2, and bronchopleural fistula in 2. Minor but serious postoperative complications included postoperative bleeding in one patient and right middle lobe torsion in another patient. All complications were resolved surgically.

Postoperative death was noted in 1 patient, and the mortality rate was 0.1%. Mortality and severe morbidities according to the RC-NCD were observed in 67 patients (11.4%); the severe morbidities were respiratory failure in 4 patients, tracheobronchial fistula in 2, pneumonia in 12, redo surgery in 2 (including postoperative bleeding in 1), arrhythmia requiring therapy in 44 (atrial fibrillation in 37, paroxysmal supraventricular tachycardia in 3, ventricular tachycardia in 3, and sick sinus syndrome in 1), and chylothorax in 2.

The median mortality rate according to the RC-NCD was 0.2%, the median mortality and severe morbidity rate was 4.6%, the median incidence rate of tracheal or bronchial fistula was 0.2%, and the median incidence rate of respiratory dysfunction was 0.2%.

### Results of the univariate analysis

The relationships between patient characteristics or operative factors and postoperative morbidity or severe morbidity were analyzed (Table 2). Postoperative morbidities were more likely to be associated with the coexistence of COPD (p = 0.03), coexistence of asthma (p < 0.01), smoking status (p < 0.01), CEA (p = 0.03), NLR > 1.56 (p = 0.01), FEV_1_% < 70 (p < 0.01), pulmonary lobe (right upper or right lower or left upper) (p < 0.01), squamous cell carcinoma (p = 0.02), and extend-lobectomy (p = 0.02) than other factors. Severe postoperative morbidity was more likely to be associated with low %VC (p < 0.02), squamous cell carcinoma (p < 0.01), and extend-lobectomy (p = 0.01) than other factors. Significant risk factors for other postoperative complications could not be assessed because of the small number of patients with these complications.

**Table 2:** Univariate analysis of postoperative morbidity and severe morbidity

Variables		Morbidity		P value	Severe morbidity		P value
		Absent, n (%)	Present, n (%)		Absent, n (%)	Present, n (%)	
Sex	Man	254 (69.8)	110 (30.2)	0.92	317 (87.1)	47 (12.9)	0.15
	Woman	155 (70.1)	66 (29.8)		201 (90.9)	20 (9.1)	
Age	≥ 75	105 (74.5)	36 (25.5)	0.17	127 (90.1)	14 (9.9)	0.51
	< 75	304 (68.5)	140 (31.5)		391 (88.1)	53 (11.9)	
Comorbidity							
Malignant disease	Absent	347 (68.8)	157 (31.2)	0.16	444 (88.1)	60 (11.9)	0.39
	Present	62 (76.5)	19 (23.5)		74 (91.4)	7 (8.6)	
Angina pectoris	Absent	386 (70.4)	162 (29.6)	0.28	484 (88.3)	64 (11.7)	0.50
	Present	23 (62.2)	14 (37.8)		34 (91.9)	3 (8.1)	
Cerebral infarction	Absent	392 (69.9)	169 (30.1)	0.92	495 (88.2)	66 (11.8)	0.25
	Present	17 (70.8)	7 (29.2)		23 (95.8)	1 (4.2)	
Interstitial lung disease	Absent	402 (69.8)	174 (30.2)	0.60	511 (88.7)	65 (11.3)	0.30
	Present	7 (77.8)	2 (22.2)		7 (77.8)	2 (22.2)	
Chronic renal failure	Absent	406 (70.2)	172 (29.8)	0.11	512 (88.6)	66 (11.4)	0.81
	Present	3 (42.8)	4 (57.2)		6 (85.7)	1 (14.3)	
Diabetes mellitus	Absent	355 (70.4)	149 (29.6)	0.49	446 (88.5)	58 (11.5)	0.91
	Present	54 (66.7)	27 (33.3)		72 (88.9)	9 (11.1)	
COPD	Absent	390 (70.9)	160 (29.1)	0.03	490 (89.1)	60 (10.9)	0.10
	Present	19 (54.3)	16 (45.7)		28 (80.0)	7 (20.0)	
Asthma	Absent	401 (71.0)	164 (29.0)	< 0.01	501 (88.7)	64 (11.3)	0.61
	Present	8 (40.0)	12 (60.0)		17 (85.0)	3 (15.0)	
Arrhythmia	Absent	395 (69.9)	170 (30.1)	0.99	500 (88.5)	65 (11.5)	0.83
	Present	14 (70.0)	6 (30.0)		18 (90.0)	2 (10.0)	
Autoimmune disease	Absent	397 (69.6)	173 (30.3)	0.38	505 (88.6)	65 (11.4)	0.81
	Present	12 (80.0)	3 (20.0)		13 (86.7)	2 (13.3)	
Hypertension	Absent	344 (69.8)	149 (30.2)	0.86	433 (87.8)	60 (12.2)	0.20
	Present	65 (70.6)	27 (29.3)		85 (92.4)	7 (7.6)	
Smoking status	Brinkman index < 600	223 (76.9)	67 (23.1)	< 0.01	263 (90.7)	27 (9.3)	0.10
	Brinkman index ≥ 600	186 (63.1)	109 (36.9)		255 (86.4)	40 (13.6)	
BMI	< 21.88	149 (67.1)	73 (32.9)	0.24	203 (91.4)	19 (8.6)	0.08
	≥ 21.88	260 (71.6)	103 (28.4)		315 (86.8)	48 (13.2)	
CEA	≤ 5	299 (72.6)	113 (27.4)	0.03	367 (89.1)	45 (10.9)	0.53
	> 5	110 (63.6)	63 (36.4)		151 (87.3)	22 (12.7)	
PNI	≥ 49.68	228 (73.3)	83 (26.7)	0.05	282 (90.7)	29 (9.3)	0.08
	< 49.68	181 (66.1)	93 (33.9)		236 (86.1)	38 (13.9)	
NLR	≤ 1.56	108 (78.3)	30 (20.0)	0.01	124 (89.9)	14 (10.1)	0.58
	> 1.56	301 (67.3)	146 (32.7)		394 (88.1)	53 (11.9)	
%VC	≥ 80	402 (70.3)	170 (29.7)	0.20	510 (89.2)	62 (10.8)	< 0.01
	< 80	7 (53.8)	6 (46.2)		8 (61.5)	5 (38.5)	
FEV_1_%	≥ 70	285 (75.4)	93 (24.6)	< 0.01	341 (90.2)	37 (8.6)	0.08
	< 70	124 (59.9)	83 (40.1)		177 (85.5)	30 (14.5)	
Pulmonary lobe of NSCLC	RUL	120 (67.0)	59 (33.0)	0.01	157 (87.7)	22 (12.3)	0.16
	RML	31 (83.8)	6 (16.2)		34 (91.9)	3 (8.1)	
	RLL	97 (70.8)	40 (29.2)		122 (89.1)	15 (10.9)	
	LUL	87 (62.6)	52 (37.4)		117 (84.2)	22 (15.8)	
	LLL	74 (79.6)	19 (20.4)		88 (94.6)	5 (5.4)	
	RUL or RLL or LUL	304 (66.8)	151 (33.2)	< 0.01	396 (87.0)	59 (13.0)	0.03
	RML or LLL	105 (80.8)	25 (19.2)		122 (93.9)	8 (6.1)	
Histological type	Non-Squamous cell carcinoma	343 (71.9)	134 (28.1)	0.02	432 (90.6)	45 (9.4)	< 0.01
	Squamous cell carcinoma	66 (61.1)	42 (38.9)		86 (79.6)	22 (20.4)	
Operative approach	VATS or RATS	362 (69.6)	158 (30.4)	0.65	464 (89.2)	56 (10.8)	0.14
	Open thoracotomy	47 (72.3)	18 (27.7)		54 (83.1)	12 (16.9)	
Operative procedure	Except for segmentectomy	356 (68.9)	161 (31.1)	0.12	452 (87.4)	65 (12.6)	0.01
	Segmentectomy	53 (77.9)	15 (22.1)		66 (97.1)	2 (2.9)	
	Except for lobectomy	78 (69.6)	34 (30.4)	0.94	97 (86.6)	15 (13.4)	0.47
	Lobectomy	331 (70.0)	142 (30.0)		421 (89.0)	52 (11.0)	
	Except for extend-lobectomy	396 (70.8)	163 (29.2)	0.02	499 (89.3)	60 (10.7)	0.01
	Extend-lobectomy	13 (50.0)	13 (50.0)		19 (73.1)	7 (26.9)	
	Except for pneumonectomy	397 (70.0)	170 (30.0)	0.76	506 (89.2)	61 (10.8)	< 0.01
	Pneumonectomy	12 (66.7)	6 (33.3)		12 (66.7)	6 (33.3)	

*COPD* chronic obstructive pulmonary disease, *BMI* body mass index, *CEA* carcinoembryonic antigen, *PNI* prognostic nutritional index, *NLR* neutrophil-to-lymphocyte ratio, *VC* vital capacity, *FEV*_*1*_*%* forced expiratory volume % in one second, *NSCLC* non-small cell lung cancer, *RUL* right upper lobe, *RML* right middle lobe, *RLL* right lower lobe, *LUL* left upper lobe, *LLL* left lower lobe, *PNI* prognostic nutritional index, *NLR* neutrophil-to-lymphocyte ratio, *VATS* video-assisted thoracic surgery, *RATS* robotic-assisted thoracic surgery

### Results of the multivariate analysis

Multivariate analyses of the risk factors for postoperative morbidities are presented in Table 3. The coexistence of asthma (odds ratio [OR] 3.07, 95% confidence interval [CI] 1.164–8.102, p = 0.02), NLR > 1.56 (OR 1.60, 95% CI 1.006–2.563, p = 0.04), and pulmonary lobe (right upper or right lower or left upper) (OR 2.01, 95%CI 1.224–3.314, p < 0.01) were identified as significant risk factors for postoperative morbidity. Furthermore, %VC < 80 (OR 6.10, 95% CI 1.888–19.712, p < 0.01), pulmonary lobe (right upper or right lower or left upper) (OR 2.27, 95%CI 1.041–4.957, p = 0.03), and extend-lobectomy (OR 3.00, 95%CI 1.201–7.493, p = 0.01) were identified as a significant risk factor for severe postoperative morbidity.

**Table 3:** Multivariate analysis of risk factors for postoperative morbidity and severe postoperative morbidity

Postoperative morbidity				
Variables		Odds ratio	95% CI	P value
Comorbidity	COPD	1.35	0.645–2.859	0.41
	Asthma	3.07	1.164–8.102	0.02
Smoking status	Brinkman index ≥ 600	1.38	0.901–2.129	0.13
CEA	> 5	1.32	0.885–1.978	0.17
FEV1%	< 70	1.49	0.984–2.257	0.05
NLR	> 1.56	1.60	1.006–2.563	0.04
Histological type	Squamous cell carcinoma	1.20	0.729–1.977	0.47
Pulmonary lobe	RUL or RLL or LUL	2.01	1.224–3.314	< 0.01
Operative procedure	Lobectomy or more	1.56	0.664–3.671	0.30
Postoperative severe morbidity				
%VC	< 80	6.10	1.888–19.712	< 0.01
Pulmonary lobe	RUL or RLL or LUL	2.27	1.041–4.957	0.03
Operative procedure	Lobectomy or more	3.00	1.201–7.493	0.01

*COPD* chronic obstructive pulmonary disease, *CEA* carcinoembryonic antigen, *FEV*_*1*_*%* forced expiratory volume % in one second, *NLR* neutrophil-to-lymphocyte ratio, *BMI* body mass index, *VC* vital capacity

### Results of the sub-analysis

We analyzed the risk factors for postoperative morbidity in patients who underwent lobectomy as a sub-analysis. The clinical characteristics of the 473 patients who underwent lobectomy are shown in Table 4. Among these, 163 underwent right upper lobectomy (RUL), 37 underwent right middle lobectomy (RML), 94 underwent right lower lobectomy (RLL), 105 underwent left upper lobectomy (LUL), and 74 underwent left lower lobectomy (LLL). Postoperative morbidities were observed in 142 patients (30.0%). A Clavien–Dindo grade I complication was noted in 1 patient, grade II in 54, grade IIIa in 83, and grade IIIb in 4. The mortality rate was 0. Mortality and severe morbidities according to the RC-NCD were observed in 52 patients (10.9%). The median mortality rate according to the RC-NCD was 0.2%, the median mortality and severe morbidity rate was 4.6%, the median incidence rate of tracheal or bronchial fistula was 0.2%, and the median incidence rate of respiratory dysfunction was 0.2%.

**Table 4:** Patient characteristics and perioperative data received lobectomy

Variables	
Sex (man / woman)	303 / 170
Age, medial, range (y)	69 (34–92)
Comorbidity	232 (53.1%)
Malignant disease	58 (12.2%)
Hypertension	73 (15.4%)
Diabetes mellitus	71 (15.0%)
Angina pectoris	32 (6.8%)
COPD	29 (6.1%)
Cerebral infarction	20 (4.2%)
Arrhythmia	16 (3.4%)
Interstitial lung disease	7 (1.5%)
Asthma	14 (2.9%)
Autoimmune disease	11 (2.3%)
Chronic renal failure	6 (1.2%)
Body mass index, median (range)	22.9 (14.3–36.6)
CEA, median, range (ng/ml)	3.4 (0.5–269)
Brinkman index, median (range)	600 (0–3600)
PNI, median (range)	50.1 (31.8–64.2)
NLR, median (range)	2.07 (0.77–12.65)
%VC, median (range)	111.2 (45.7–184.2)
FEV1%, median (range)	73.8 (30.5–99.5)
pStage (0 / IA / IB / IIA / IIB / IIIA / IIIB / IV / yIA)	16 / 234 / 97 / 39 / 43 / 36 / 3 / 2 / 3
Histological type (Ad / Sq / LCNEC / AdSq / Pleo / Large / Carci)	362 / 81 / 14 / 8 / 2 / 3 / 3
Operative approach (Open / VATS / RATS)	48 / 419 / 6
Operative procedure	
RUL	163 (34.5%)
RML	37 (7.8%)
RLL	94 (19.8%)
LUL	105 (22.2%)
LLL	74 (15.6%)
Morbidity	142 (30.0%)
Air leakage	67 (14.1%)
Arrhythmia	35 (7.4%)
Atelectasis	16 (3.4%)
Pneumonia	8 (1.7%)
Attack of asthma	3 (0.6%)
Cerebral infarction	3 (0.6%)
Chylothorax	2 (0.4%)
Broncho-pleural fistula	1 (0.2%)
Clavien–Dindo grade (0 / 1 / 2 / 3a / 3b)	331 / 1 / 54 / 83 / 4
Mortality	0 (0%)
Mortality or severe morbidity	52 (10.9%)
Tracheal or bronchial fistula	1 (0.2%)
Respiratory disfunction	4 (0.8%)
NCD risk score	
Mortality, median (range) (%)	0.2 (0–10.3)
Mortality or severe morbidity, median (range) (%)	4.6 (1.2–27.3)
Tracheal or bronchial fistula, median (range) (%)	0.2 (0–3.4)
Respiratory disfunction, median (range) (%)	0.2 (0–4.4)

*COPD* chronic obstructive pulmonary disease, *CEA* carcinoembryonic antigen, *PNI* prognostic nutritional index, *NLR* neutrophil-to-lymphocyte ratio, *VC* vital capacity, *FEV*_*1*_*%* forced expiratory volume % in one second, *p* pathological, *y* yield to treatment, *Ad* adenocarcinoma, *Sq* squamous cell carcinoma, *LCNEC* large cell neuroendocrine carcinoma, *AdSq* adenosquamous cell carcinoma, *Large* large cell carcinoma, *Carci* carcinoid, *Open* open thoracotomy, *VATS* video-assisted thoracic surgery, *RATS* robotic-assisted thoracic surgery *RUL* right upper lobectomy, *RML* right middle lobectomy, *RLL* right lower lobectomy, *LUL* left upper lobectomy, *LLL* left lower lobectomy, *NCD* national clinical database

The comparison between the risk score according to the RC-NCD and postoperative morbidity by lobes is shown in Table 5. Although the risk scores for mortality or severe morbidity according to the RC-NCD were extremely high in RUL and RLL, the mortality and severe morbidity rates were not significantly different among lobes, although the morbidity rate tended to be high in RUL, RLL, and LUL.

**Table 5:** Comparison between risk score according to RC-NCD and postoperative morbidity by lobes

Procedure	RUL	RML	RLL	LUL	LLL	P value
NCD risk score						
Mortality, median (range) (%)	0.3 (0–2.5)	0.1 (0–1.1)	0.4 (0–10.3)	0.1 (0–2.4)	0.1 (0–4.3)	< 0.01
Mortality or severe morbidity, median (range) (%)	5.7 (1.8–19.7)	2.6 (1.2–12.2)	5.3 (1.8–27.3))	3.7 (1.2–21.6)	2.9 (1.2–16.5)	< 0.01
Tracheal or bronchial fistula, median (range) (%)	0.2 (0–0.6)	0.1 (0–0.8)	0.6 (0.1–3.4)	0.2 (0–0.6)	0.1 (0–1.1)	< 0.01
Respiratory disfunction, median (range) (%)	0.2 (0–1.4)	0.1 (0–0.7)	0.2 (0–2.5)	0.2 (0–2.5)	0.1 (0–4.4)	< 0.01
Clinical data						
Mortality or severe morbidity (%)	11.6	8.1	9.6	17.1	4.1	0.08
Tracheal or bronchial fistula (%)	0	0	0	0.9	0	0.47
Respiratory disfunction (%)	0.6	0	1.1	1.9	0	0.64
Morbidity (%)	32.5	16.2	32.9	35.2	20.3	0.06
Air leakage (%)	14.7	5.4	18.1	14.3	12.1	0.43
Arrhythmia (%)	7.9	8.1	5.3	11.4	2.7	0.23
Pneumonia (%)	1.2	0	2.1	2.8	1.3	0.76
Atelectasis (%)	4.9	2.7	3.2	1.9	2.7	0.73

*RC-NCD* risk calculator of national clinical database, *RUL* right upper lobectomy, *RML* right middle lobectomy, *RLL* right lower lobectomy, *LUL* left upper lobectomy, *LLL* left lower lobectomy

The relationships between patient characteristics or operative factors and postoperative morbidity or severe morbidity for patients who underwent lobectomy were analyzed (Table 6). Postoperative morbidities tended to be associated with the coexistence of chronic renal failure (p = 0.04), coexistence of asthma (p < 0.04), smoking status (p < 0.01), CEA (p = 0.04), NLR > 1.56 (p = 0.01), FEV_1_% < 70 (p < 0.01), and RUL, RLL, or LUL (p < 0.01). Severe postoperative morbidity tended to be associated with male sex (p = 0.01), a high BMI (p < 0.01), low %VC (p < 0.02), squamous cell carcinoma (p = 0.04), and RUL, RLL, or LUL (p = 0.03).

**Table 6:** Univariate analysis of risk factors for postoperative morbidity and severe morbidity in patients received lobectomy

Variables		Morbidity		P value	Severe morbidity		P value
		Absent, n (%)	Present, n (%)		Absent, n (%)	Present, n (%)	
Sex	Man	209 (68.9)	94 (31.1)	0.52	262 (86.5)	41 (13.5)	0.01
	Woman	122 (71.8)	48 (28.2)		159 (93.5)	11 (6.5)	
Age	≥ 75	77 (72.6)	29 (27.4)	0.49	94 (88.7)	12 (11.3)	0.90
	< 75	254 (69.2)	113 (30.8)		327 (89.1)	40 (10.9)	
Comorbidity							
Malignant disease	Absent	288 (69.4)	127 (30.6)	0.46	369 (88.9)	46 (11.1)	0.86
	Present	43 (74.1)	15 (25.9)		52 (89.7)	6 (10.3)	
Angina pectoris	Absent	311 (70.5)	130 (29.5)	0.33	392 (88.9)	49 (11.1)	0.76
	Present	20 (62.5)	12 (37.5)		29 (90.6)	3 (9.4)	
Cerebral infarction	Absent	318 (70.2)	135 (29.8)	0.61	402 (88.7)	51 (11.3)	0.38
	Present	13 (65.0)	7 (35.0)		19 (95.0)	1 (5.0)	
Interstitial lung disease	Absent	325 (69.7)	141 (30.3)	0.36	415 (89.1)	51 (10.9)	0.77
	Present	6 (85.7)	1 (14.3)		6 (85.7)	1 (14.3)	
Chronic renal failure	Absent	329 (70.5)	138 (29.5)	0.04	416 (89.1)	51 (10.9)	0.65
	Present	2 (33.3)	4 (66.7)		5 (83.3)	1 (16.7)	
Diabetes mellitus	Absent	284 (70.7)	118 (29.3)	0.45	358 (89.1)	44 (10.1)	0.93
	Present	47 (66.2)	24 (33.8)		63 (88.7)	8 (11.3)	
COPD	Absent	315 (70.9)	129 (29.1)	0.07	398 (89.6)	46 (10.4)	0.08
	Present	16 (55.2)	13 (44.8)		23 (79.3)	6 (20.7)	
Asthma	Absent	326 (71.0)	133 (29.0)	< 0.01	410 (89.3)	49 (10.7)	0.20
	Present	5 (35.7)	9 (64.3)		11 (78.6)	3 (21.3)	
Arrhythmia	Absent	321 (70.2)	136 (29.8)	0.50	407 (89.61	50 (10.9)	0.84
	Present	10 (62.5)	6 (37.5)		14 (87.5)	2 (12.5)	
Autoimmune disease	Absent	323 (69.9)	139 (30.1)	0.84	412 (89.2)	50 (10.8)	0.44
	Present	8 (72.7)	3 (27.3)		9 (81.8)	2 (18.2)	
Hypertension	Absent	279 (69.7)	121 (30.3)	0.79	354 (88.5)	46 (11.5)	0.40
	Present	52 (71.2)	21 (28.8)		67 (91.8)	6 (8.2)	
Smoking status	Brinkman index < 600	180 (76.3)	56 (23.7)	< 0.01	213 (90.3)	23 (9.7)	0.38
	Brinkman index ≥ 600	151 (63.7)	86 (36.3)		208 (87.8)	29 (12.2)	
BMI	< 22.93	165 (70.8)	68 (29.2)	0.69	217 (93.2)	16 (6.8)	< 0.01
	≥ 22.93	166 (69.2)	74 (30.8)		204 (85.0)	36 (15.0)	
CEA	≤ 5	240 (72.7)	90 (27.3)	0.04	297 (90.0)	33 (10.0)	0.29
	> 5	91 (63.6)	52 (36.4)		124 (86.7)	19 (13.3)	
PNI	≥ 49.68	190 (73.9)	67 (26.1)	0.04	234 (91.1)	23 (8.9)	0.12
	< 49.68	141 (65.3)	75 (34.7)		187 (86.6)	29 (13.4)	
NLR	≤ 1.56	97 (78.2)	27 (21.8)	0.01	111 (89.5)	13 (10.5)	0.83
	> 1.56	234 (67.1)	115 (32.9)		310 (88.8)	39 (11.2)	
%VC	≥ 80	325 (70.4)	137 (29.6)	0.25	414 (89.6)	48 (10.4)	< 0.01
	< 80	6 (54.6)	5 (45.4)		7 (63.6)	4 (36.4)	
FEV_1_%	≥ 70	231 (74.3)	80 (25.7)	< 0.01	281 (90.4)	30 (9.6)	0.19
	< 70	100 (61.7)	62 (38.3)		140 (86.4)	22 (13.6)	
Histological type	Non-Squamous cell carcinoma	279 (71.2)	113 (28.8)	0.21	354 (90.3)	38 (9.7)	0.04
	Squamous cell carcinoma	52 (64.2)	29 (35.8)		67 (82.7)	14 (17.3)	
Operative approach	VATS or RATS	295 (69.4)	130 (30.6)	0.42	380 (89.4)	45 (10.6)	0.40
	Open thoracotomy	36 (75.0)	12 (25.0)		41 (85.4)	7 (14.6)	
Operative procedure	RML or LLL	90 (81.1)	21 (18.9)	< 0.01	105 (94.6)	6 (5.4)	0.03
	RUL or RLL or LUL	241 (66.6)	121 (33.4)		316 (87.3)	46 (12.7)	

*COPD* chronic obstructive pulmonary disease, *CEA* carcinoembryonic antigen, *PNI* prognostic nutritional index, *NLR* neutrophil-to-lymphocyte ratio, *VC* vital capacity, *FEV*_*1*_*%* forced expiratory volume % in one second, *VATS* video-assisted thoracic surgery, *RATS* robotic-assisted thoracic surgery, *RML* right middle lobectomy, *LLL* left lower lobectomy, *RUL* right upper lobectomy, *RLL* right lower lobectomy, *LUL* left upper lobectomy

Multivariate analyses of risk factors for postoperative morbidity and severe morbidity for patients who underwent lobectomy are shown in Table 7. The coexistence of asthma (OR 4.18, 95% CI 1.291–13.556, p = 0.01) and the performance of RUL, RLL, or LUL (OR 2.21, 95% CI 1.277–3.823, p < 0.01) were identified as significant risk factors for postoperative morbidity, while male sex (OR 2.35, 95% CI 1.152–4.818, p = 0.01), a high BMI (OR 2.41, 95% CI 1.277–4.550, p < 0.01), low %VC (OR 4.09, 95% CI 1.034–16.174, p = 0.04), and performance of RUL, RLL, or LUL (OR 2.65, 95% CI 1.083–6.495, p = 0.03) were identified as significant risk factors for severe postoperative morbidity.

**Table 7:** Multivariate analysis of risk factors for postoperative morbidity and severe morbidity in patients received lobectomy

Postoperative morbidity				
Variables		Odds ratio	95% CI	P value
Comorbidity	Chronic renal failure	4.82	0.809–28.755	0.08
	Asthma	4.18	1.291–13.556	0.01
Smoking status	Brinkman index ≥ 600	1.45	0.930–2.266	0.10
CEA	> 5	1.37	0.878–2.156	0.16
PNI	< 49.68	1.40	0.921–2.130	0.11
NLR	> 1.56	1.49	0.903–2.477	0.11
FEV1%	< 70	1.30	0.828–2.051	0.25
Operative procedure	RUL or RLL or LUL	2.21	1.277–3.823	< 0.01
*Postoperative severe morbidity*				
Sex	Man	2.35	1.152–4.818	0.01
BMI	≥ 22.93	2.41	1.277–4.550	< 0.01
%VC	< 80	4.09	1.034–16.174	0.04
Histology	Squamous cell carcinoma	1.66	0.819–3.368	0.15
Operative procedure	RUL or RLL or LUL	2.65	1.083–6.495	0.03

*CEA* carcinoembryonic antigen, *PNI* prognostic nutritional index, *NLR* neutrophil-to-lymphocyte ratio, *FEV*_*1*_*%* forced expiratory volume % in one second, *RUL* right upper lobectomy, *RLL* right lower lobectomy, *LUL* left upper lobectomy, *RUL* right upper lobectomy, *RLL* right lower lobectomy, *LUL* left upper lobectomy, *BMI* body mass index, *VC* vital capacity

## Discussion

In the present study, we analyzed the risk factors for postoperative morbidity in patients who underwent pulmonary resection for NSCLC. Several factors, such as the age, comorbidity, smoking history, operative approach, and type of operative procedure, have been reported as risk factors for postoperative morbidity in NSCLC patients who have undergone pulmonary resection [5, 7, 8, 13, 20, 21]. The sex, coexistence of asthma, FEV_1_%, and type of operative procedure were shown to be significant risk factors for postoperative morbidity in a previous study [22]; in the present study as well, the coexistence of asthma, NLR, and pulmonary lobe were significant risk factors for postoperative morbidity, whereas the %VC, pulmonary lobe and type of operative procedure were significant risk factors for severe postoperative morbidity.

RLL has been reported to be a significant risk factor for severe postoperative morbidity in the Japanese nationwide database [12]. Although the risk factors for postoperative morbidity might vary depending on the number of patients or patient characteristics, the type of operative procedure is likely to be a significant risk factor for postoperative morbidity in patients who have undergone pulmonary resection. In the present study, the mortality and severe morbidity rates were not significantly different among lobes, whereas the morbidity rate tended to be high in RUL, RLL, and LUL. Furthermore, the operative procedure, including RUL, RLL, and LUL, was a significant risk factor for severe postoperative morbidity in the multivariate analysis. Therefore, we considered that the RC-NCD for postoperative severe morbidity should need to be modified according to high-risk lobes.

In the present study, postoperative morbidity for patients who underwent lobectomy was significantly related to the coexistence of asthma and the type of operative procedure, specifically RUL, RLL, or LUL. Although air leakage requiring therapy, atelectasis, asthma attacks, and cerebral infarction are not considered severe postoperative morbidities by the RC-NCD, they are important complications after pulmonary resection. In a previous study, the sex and operative approach, including VATS and RATS, were identified as significant risk factors for postoperative air leakage, while the coexistence of asthma was a significant risk factor for postoperative atelectasis [22]. Cerebral infarction after pulmonary resection is a rare but unfortunate complication, and one risk factor that has been analyzed and reported was the performance of LUL [23]. Cerebral infarction is considered to increase due to blood clots in pulmonary vein stump. Because cerebral infarction is rare but an important postoperative complication, the risk analysis for postoperative cerebral infarction should be done separately in RC-NCD. On the other hand, air leakage was most morbidity in the present study. Furthermore, atelectasis and attack of asthma was rare but the important morbidities. Therefore, air leakage requiring therapy, atelectasis, and asthma attacks should be included as important postoperative morbidities in the RC-NCD. Furthermore, the risk factors related to these important postoperative morbidities after pulmonary resection should be analyzed using a large-scale survey in the future.

Several limitations associated with the present study warrant mention. For example, this study had a retrospective design, patients' performance status wasn't analyzed, and there was a possibility of unobserved confounding and selection bias. In addition, the present study was performed at a single institution.

## Conclusions

We analyzed the risk factors for postoperative morbidity in patients who underwent pulmonary resection. The coexistence of asthma, NLR, and pulmonary lobe were significant risk factors for postoperative morbidity in the present study, and the %VC, pulmonary lobe, and type of operative procedure were significant risk factors for severe postoperative morbidity. Furthermore, in patients received lobectomy, coexistence of asthma and pulmonary lobe were identified as significant risk factors for postoperative morbidity, meanwhile, male sex, high BMI, low VC, and pulmonary lobe were identified as significant risk factors for severe postoperative morbidity. Given that the pulmonary lobe was a significant risk factor for postoperative morbidity in patients received pulmonary resection and for severe postoperative morbidity in patients received lobectomy, the RC-NCD for postoperative morbidity needs to be modified according to high-risk lobes.

## Data Availability

The datasets generated and/or analyzed during the current study are not publicly available due to [our institutional restrictions e.g., them containing information that could compromise research participant privacy/consent], but are available from the corresponding author on reasonable request.

## Abbreviations

NSCLC: Non-small cell lung cancer
COPD: Chronic obstructive pulmonary disease
RC-NCD: Risk calculator of national clinical database
BMI: Body mass index
CEA: Carcinoembryonic antigen
PNI: Prognostic nutritional index
NLR: Neutrophil-to-lymphocyte ratio
CT: Computed tomography
VC: Vital capacity
FEV1%: Forced expiratory volume % in one second
VATS: Video-assisted thoracic surgery
RATS: Robotic-assisted thoracic surgery
OR: Odds ratio
CI: Confidence interval
RUL: Right upper lobectomy
RML: Right middle lobectomy
RLL: Right lower lobectomy
LUL: Left upper lobectomy
LLL: Left lower lobectomy
